# A possible founder mutation in *FZD6* gene in a Turkish family with autosomal recessive nail dysplasia

**DOI:** 10.1186/s12881-019-0746-6

**Published:** 2019-01-14

**Authors:** Ceren Saygı, Yasemin Alanay, Uğur Sezerman, Aslı Yenenler, Nesrin Özören

**Affiliations:** 1 Department of Molecular Biology and Genetics, Boğaziçi University, Istanbul, Turkey; 2 Pediatric Genetics Unit, Department of Pediatrics, School of Medicine, Acibadem Mehmet Ali Aydinlar University, Istanbul, Turkey; 3 Department of Medical Statistics and Bioinformatics, School of Medicine, Acibadem Mehmet Ali Aydinlar University, Istanbul, Turkey

**Keywords:** FZD6, Whole exome sequencing, Consanguinity, Autosomal recessive, Nail dysplasia, Turkey

## Abstract

**Background:**

Autosomal recessive nail dysplasia is characterized by thick and hard nails with a very slow growth on the hands and feet. Mutations in *FZD6* gene were found to be associated with autosomal recessive nail dysplasia in 2011. Presently, only seven mutations have been reported in *FZD6* gene; five mutations are clustered in the C-terminus, one is at the seventh transmembrane domain, and another is at the very beginning of third extracellular loop.

**Methods:**

Whole exome sequencing (WES) was applied to the index case, her one affected sister and her healthy consanguineous parents. The mutation was verified via Sanger sequencing. Molecular dynamics simulations of the predicted structures of native and mutant proteins were compared to gain insight into the pathogenicity mechanism of the mutation.

**Results:**

Here, we report a homozygous 8 bp deletion mutation, p.Gly559Aspfs*16; c.1676_1683delGAACCAGC, in *FZD6* gene which causes a frameshift and creates a premature stop codon at position 16 of the new reading frame. Our molecular dynamics calculations predict that the pathogenicity of this frameshift mutation may be caused by the change in entropy of the protein with negative manner, disturbing the C-terminal domain structure, and hence interaction partners of FZD6.

**Conclusion:**

We identified a homozygous deletion mutation in *FZD6* in a consanguineous Turkish family with nail dysplasia. We also provide a molecular mechanism about the effects of the deletion on the protein structure and its possible motions. This study provides a pathogenicity mechanism for this mutation in nail dysplasia for the first time.

**Electronic supplementary material:**

The online version of this article (10.1186/s12881-019-0746-6) contains supplementary material, which is available to authorized users.

## Background

The development of human nails starts around the ninth week of gestation and is completed during the fifth month of pregnancy [1]. Human hereditary nail disorders are divided into 10 different subtypes (Nail disorder, nonsyndromic congenital 1–10; NDNC 1–10; OMIM 161050, 149,300, 151,600, 206,800, 164,800, 107,000, 605,779, 607,523, 614,149, 614,157). They constitute an extremely rare and heterogeneous group of ectodermal dysplasia and occur as isolated and/or syndromic ectodermal conditions, where other ectodermal appendages are also involved. Five genes have been found to be associated with nail dysplasia thus far; namely *HPGD*, *RSPO4*, *PLCD1*, *COL7A1* and *FZD6* [2]. Even though considerable advances have been achieved in the diagnosis and management of nail disorders, the knowledge of the molecular developmental pathways of nail growth and morphogenesis is still relatively limited.

In 2011, Fröjmark et al. were the first to identify the mutations in *FZD6* gene as a cause of autosomal recessive nail dysplasia (NDNC10, OMIM 614157). They reported two consanguineous Pakistani families with 11 members affected by isolated nail dysplasia [2, 3]. According to the study of Fröjmark et al., the homozygous *FZD6* mutations (p.Glu584* and p.Arg511Cys) result in dysfunctional FZD6/loss of FZD6 followed by a subsequent misregulation of several FZD6-mediated pathways required for the formation and regeneration of nails in a proper manner. To date, seven different mutations have been reported in eleven families, including two missense, two nonsense, two frameshifts and one compound heterozygous mutation (Additional file 1: Table S1). Five of these seven mutations are clustered in the C-terminus which suggests that the C-terminal region could be a mutation hotspot. The discovered variants include amino acid substitutions in highly conserved residues and nonsense/frameshift variants leading to signaling disruption in the C-terminus cytoplasmic domain [3–8].

**Fig. 1:**
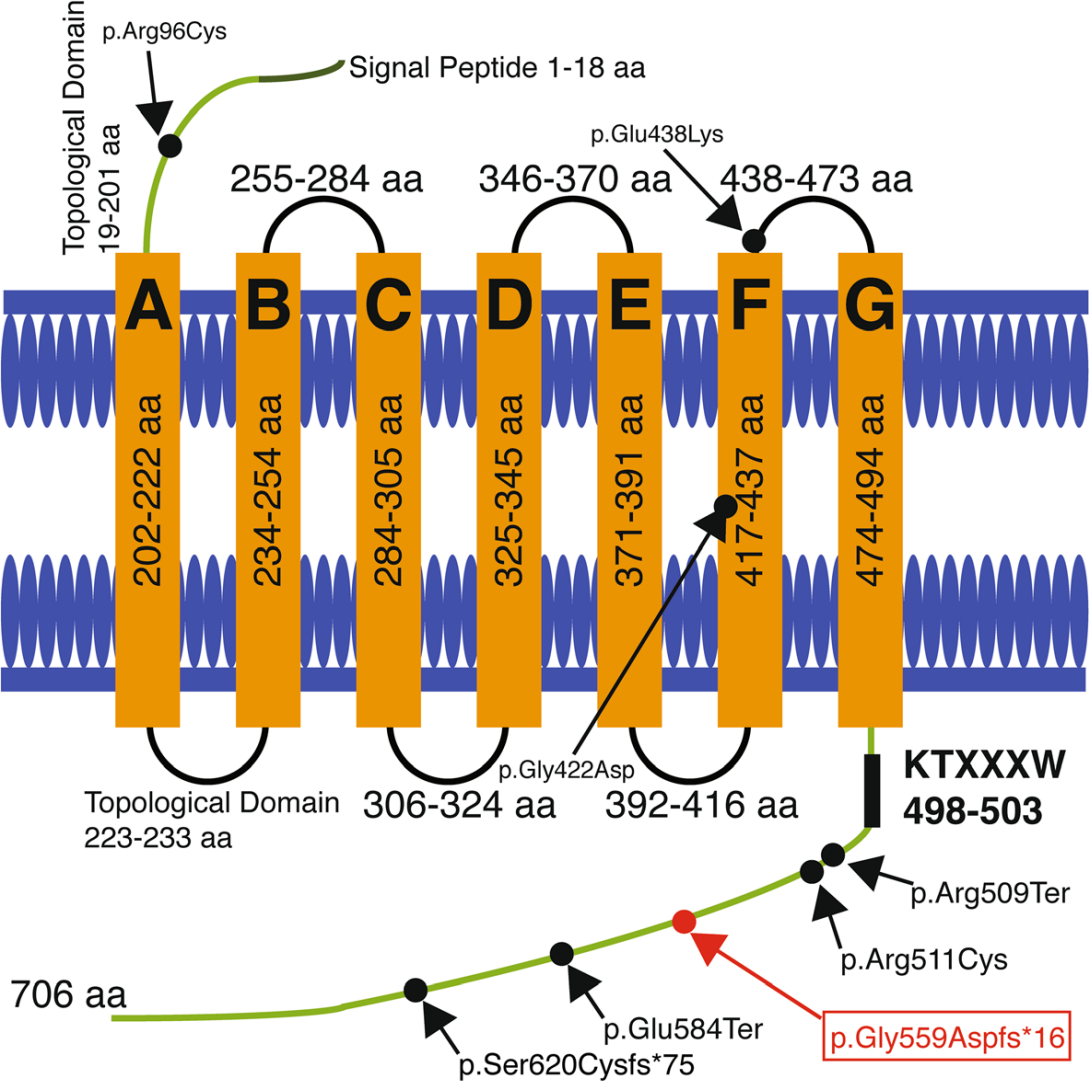
The Schematic Representation of Reported Mutations in the Literature

FZD6 is composed of seven transmembrane domains (amino acids 202–222, 234–254, 284–305, 325–345, 371–391, 417–437, 474–494) and seven topological domains (amino acids 19–201, 223–233, 255–284, 306–324, 346–370, 392–416, 438–473) (Fig. 1). It belongs to the frizzled family and, in general, frizzled family proteins expose their large N-terminus on the extracellular side containing a cysteine-rich domain (CRD) that binds the receptor’s ligands [9, 10]. All known interaction partners bind the extracellular CRD of FZD proteins [11–16]. Even though this domain is necessary for ligand binding, it is not known to be necessary for signal transduction [17]. Mutagenesis studies have revealed that there are several residues in the intracellular loops and in the C-terminal domain that are very critical for signaling [18].

Here, a consanguineous Turkish family with three affected individuals having homozygous 8 bp deletion mutation, p.Gly559Aspfs*16; c.1676_1683delGAACCAGC were reported. This amino acid change creates a premature stop codon at position 16 of the new reading frame where 133 amino acid is lost in C-terminus compared to native protein. Previously, our mutation has been reported in two other Turkish families with NDNC10 [8], and therefore, this is the third Turkish family with the same mutation, indicating that all three families have a common ancestor.

## Methods

All genetic studies were carried out after appropriate ethics approval. All participants provided written informed consent.

### NGS analysis

Informed consent was received from participants prior to inclusion in this study. Peripheral blood samples were collected from six family members following the informed consent and IRB approval (ATADEK 2017–2/17). Genomic DNA was isolated from leukocytes by standard procedures.

Captured libraries were loaded onto the HiSeq 2500 platform (Illumina). Trimmomatic was used to remove adapters, low quality (Phred quality score < 5) bases from the 3′ ends of sequence reads (Bolger, Lohse, & Usadel, 2014). Reads shorter than 36 bp were subsequently removed. Further processing was performed following the Genome Analysis Toolkit’s (GATK) best practice recommendations. Briefly, trimmed reads were aligned to the human reference genome (UCSC GRCh37/hg19) using the Burrows-Wheeler Aligner (BWA mem v0.7.12). Duplicate reads were marked with Picard tools (v1.141). GATK (v3.4) was used for indel realignment, base quality score recalibration, calling variants using the HaplotypeCaller, joint genotyping, and variant quality score recalibration. AnnoVar (v2015-03-22) was used to functionally annotate and filter alterations against public databases (dbSNP138, 1000 Genomes Project, and ExAC Browser).

### Variant filtration

One of the clearest proof to consider a variant as benign is its high allele frequency in the human population, being too high for causation of a disease [19, 20]. Hence, from variants with a MAF of < 0.1% and without homozygous carriers in public databases, the ones predicted to affect protein coding were analyzed. For the intronic alterations, the ones at exon-intron boundaries from − 10 bases to + 10 bases are retained. If no prominent variant is found, the threshold is increased to − 40 to + 40. Then, we prioritize variant lists and start with the list that matches the most expected segregation pattern. Symptoms of affected individuals and family history were reviewed to prioritize variants with the highest degree of symptom match. Evidence from various sources; population databases, computational assessments, PubMed, OMIM and MGI were gathered. Seven different tools are being considered for pathogenicity estimations; namely CADD, REVEL, M-CAP, PrimatAI [21], SIFT, Polyphen, and MutationTaster. We also investigated the effect of the splice site mutations via four different splice site prediction programs; Human Splicing Finder, NetGene2Server, Berkeley Drosophila Genome Project-Splice Site Prediction by Neural Network and Oriel SpliceView. Instead of expecting the support for the disease-causing effect of the variant from all of the in silico tools, the information obtained from each tool is taken into account; since each tool has several varying strengths and weaknesses.

### Variant validation and segregation study

Sequence validation and segregation analysis for the *FZD6* variant was performed by Sanger-sequencing. Detailed primer sequences and PCR conditions are available upon request. Sequence electropherograms were analyzed using the FinchTV (Geospiza, USA). Mutation nomenclature refers to GenBank mRNA reference sequence NM_001317796.

### 3D structure modeling

Based on the NGS sequencing results, the protein sequence containing our mutant (p.Gly559Aspfs*16; c.1676_1683delGAACCAGC.), called as FZD6 mutant, was translated via Ensembl Tool. Through I-TASSER, the structure of FZD6 mutant was predicted, and the best model among generated several ones was selected based on C-score [22, 23]. All molecular dynamics (MD) simulations were performed with NAMD program, where CHARMM 36 all-atom force fields including the correction maps were used [24–27]. Water molecule was described as TIP3P model [28]. Before production simulations, FZD6 mutant was subjected to 40,000-step minimization performed with Greedy algorithm, and it was followed by 2 ns equilibration, collected as NpT ensemble at 298 K. During simulations, periodic boundary conditions were applied at all dimensions, the systems were run with 2-fs velocity, and Langevin pressure coupling was used to keep pressure constant at 1 atm. Particle-mesh Ewald (PME) method was used to calculate the electrostatic interactions [29, 30]. Production simulations of FZD6 and FZD6 mutant were collected at 310 K as 20 ns followed by the minimization of ionized systems with the Greedy algorithm and NpT equilibration performed at 298 K with 2-fs step velocity. All MD simulations were run twice by changing their initial coordinates to avoid any artifact. The native and mutant systems were prepared to MD simulation, e.g., solvation and ionization, with Visual Molecular Dynamics (VMD). Also, the visualization and analysis of systems, e.g., Root-Mean-Square Deviation (RMSD) and Root-Mean-Square Fluctuation (RMSF) calculations, and salt bridge interactions were performed with VMD [31].

## Results

### Patients and phenotypic characteristics

**Fig. 2:**
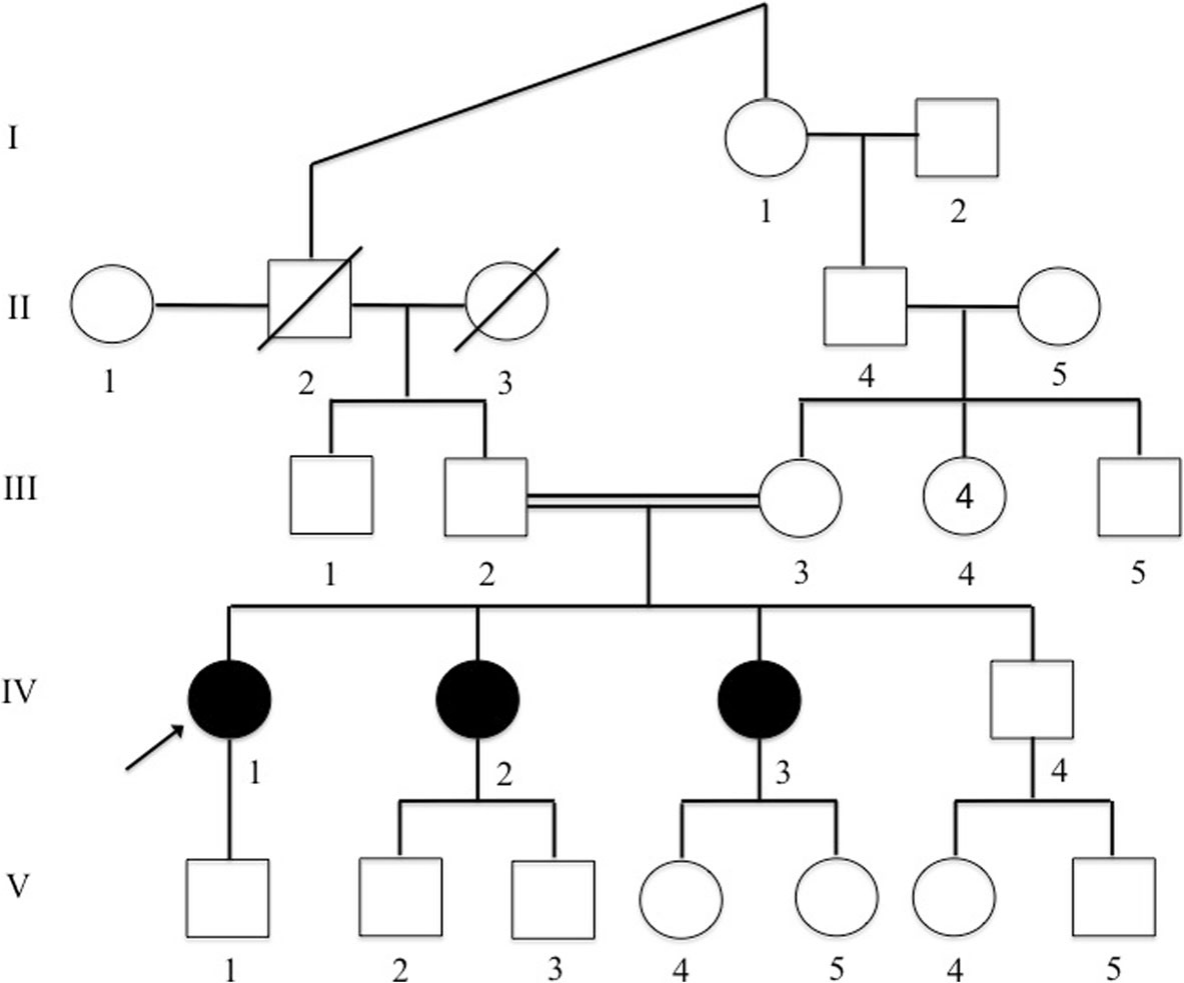
Pedigree of a consanguineous Turkish family segregating autosomal recessive isolated nail dysplasia. Circles and squares represent females and males, respectively. Clear symbols represent unaffected individuals while filled symbols represent affected individuals

The index patient is a thirty-three year old female. Her parents are first cousins. She was diagnosed with congenital nail dysplasia at birth. She was referred for genetic counselling during her first pregnancy. She had thickened, hard, shiny, hyperplastic and hyperpigmented, claw-shaped (onycholysis) nails on the hands and feet. All nails in all four extremities were affected. Intermitently she loses her nails and the newly grown ones are similarly affected. They become hard, thickened and claw-shaped in time. Pedigree analysis demonstrated two additional affected sisters and a healthy brother with parental consanguinity (Fig. 2). The patient and her sisters did not give consent for publishing of images. Her sisters were also recognized to have nail dysplasia from birth. Photographic images were examined and they suggested an identical phenotype. Both sisters were married with unrelated partners and had healthy children. The patient was not related to her partner. Analysis of the pedigree suggested autosomal recessive inheritance pattern. Therefore, recurrence of the phenotype was considered low. She was counseled accordingly. She later gave birth to a healthy boy. Postpartum she was diagnosed with uveitis and treated for ocular tuberculosis without pulmonary involvement. Her treatment regimen included 9 months of anti-tuberculosis agents. Corticosteroids were also used for the first 2 months.

Using whole-exome sequencing, 96 de novo heterozygous, 421 homozygous variants were obtained. After the population frequency and splice site filtering, as explained in the methods section, quantity decreased to 19, 46, 3 for de novo heterozygous, homozygous, compound heterozygous variants respectively. After recruiting data for gene intolerance, mouse phenotypes, pathogenicity scores, and PrimatAI; the *FZD6* gene was found to be the most prominent gene that can be associated with the disease, even though the mutation does not have a high intolerance or pathogenicity prediction scores, due to its known association with NDNC10.

The index case is found to be homozygous for an 8 bp deletion mutation, p.Gly559Aspfs*16; c.1676_1683delGAACCAGC, in *FZD6* (Fig. 2). This mutation causes a frameshift and creates a premature stop codon at position 16 of the new reading frame. The unaffected parents of each child are heterozygous carriers for this mutation. This mutation has previously been reported in two other Turkish families, indicating a common ancestor. The mutation was confirmed via Sanger sequencing. Both parents and a healthy brother are found to be heterozygous carriers while the index case and two affected sisters are homozygous.

**Fig. 3:**
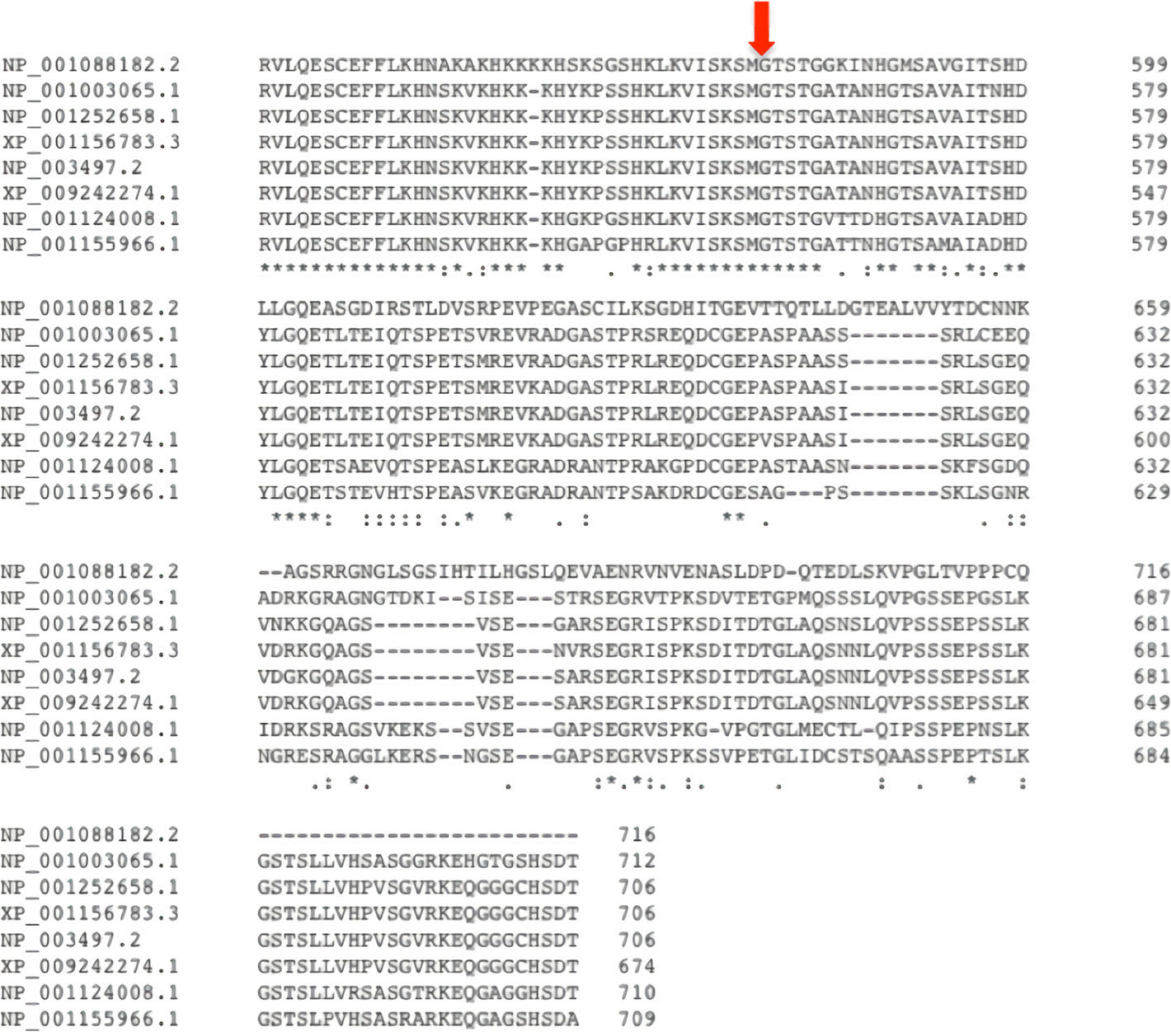
Partial amino acid sequence of the human FZD6 protein in comparison with orthologues from other species. The mutation point of c.1676_1683delGAACCAGC frameshift deletion is indicated by an arrow. Species abbreviations are as follows: Hs, *Homo sapiens*; Pt, *Pan troglodytes*; Ma m, *Macaca mulatta*; Pa, Pongo abelii; Bt, *Bos taurus,* cf*, Canis lupus familiaris*; Rn, *Rattus norvegicus*; Mm, *Mus musculus*; Xl *Xenopus laevis*. The accession numbers for the respective proteins are as follows: Hs, NP_003497.2; Pt, XP_001156717.1; Mm, NP_032082.2; Pa, XP_009242274.1; Cf, NP_001003065.1; Rn, NP_001124008.1; Mm, NP_032082.2; Xl, NP_001088182.2

Evolutionary conservation of the disappeared amino acid region in other FZD6 orthologues was examined using Clustal Omega [32]. The potential functional importance of the frameshift mutation is supported by the fact that the region is quite conserved in several species including human, rat, and mouse (Fig. 3). The Clustal Omega results of the paralogs of FZD6 suggest that both the N- and C- terminal regions are highly variable compared to the transmembrane domains (Additional file 1: Figure S2). In the light of this fact, we deduce that there is an alteration in the interactions partners and thus of variations of functions in FZD family proteins.

Up to now, ten FZDs have been identified in *Homo sapiens* [33]*.* Interestingly, C-terminus of FZD3 and FZD6 are considerably longer than those of other Frizzleds (Additional file 1: Figure S2).

### 3D structural modeling

**Fig. 4:**
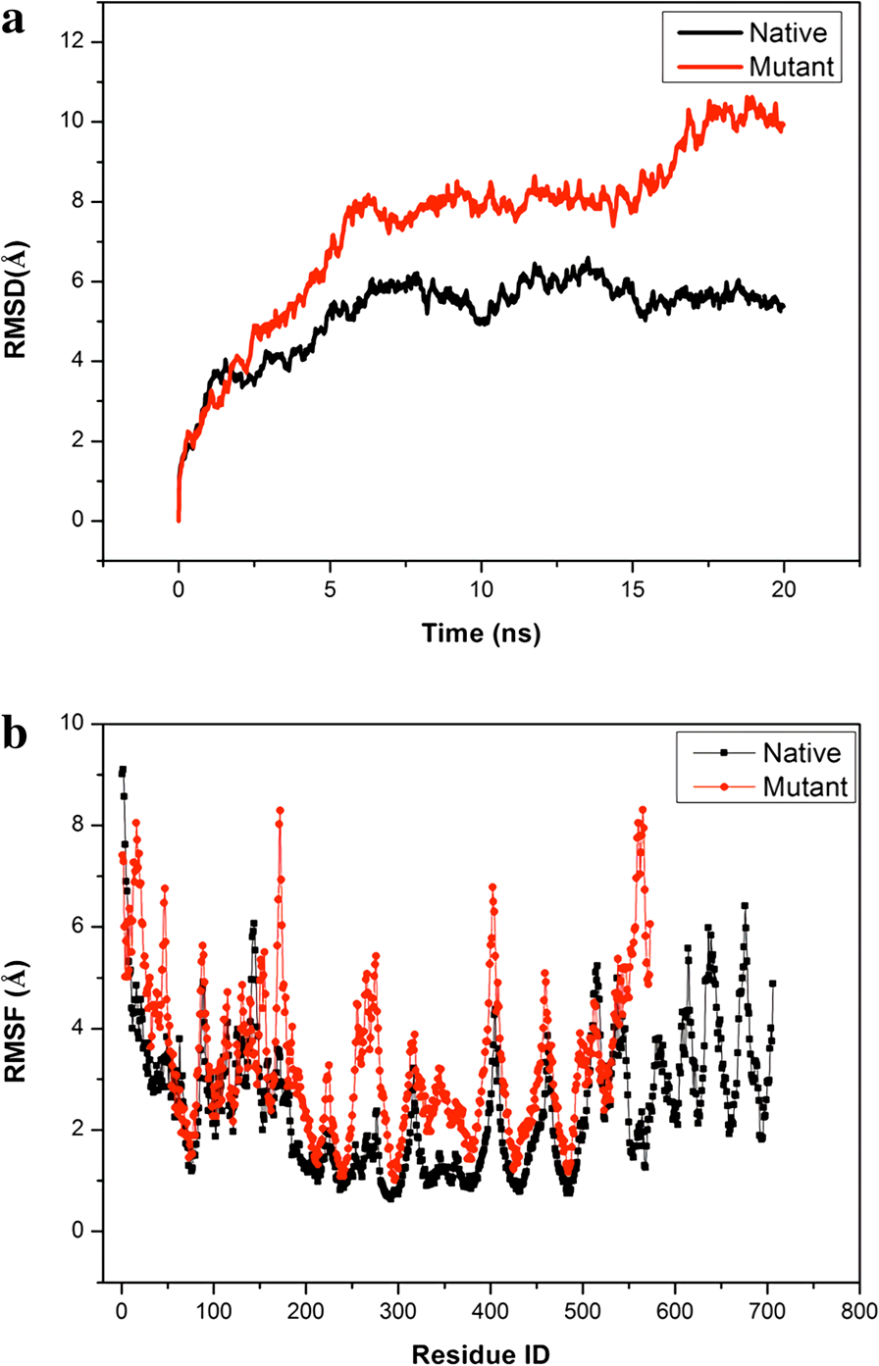
RMSD (**a**) and RMSF (**b**) results of modeled FZD6 proteins, native and mutant, along 20 ns

As stated in introductory part, the crystal structure of the FZD6 protein has not yet been deposited to Protein Data Bank (PDB). Thus, 3D structures of FZD6, including native form and p.Gly559Aspfs*16 mutation were modeled via I-TASSER before performing 20 ns MD simulations. As displayed in Fig. 4a, mutant protein displays higher backbone motion compared to native one. From the beginning of 4th ns through the end of MD trajectory, the tendency for increased RMSD is clearly observed and the particular differences in backbone responses of native and mutant ones become more apparent within last 5 ns. As in line with RMSD patterns, higher RMSF values are observed in the FZD6 mutant. Especially, Leu253-Cys282, Ala329-Phe380 and His549-Ser571 regions display higher RMSF values than the native protein (Fig. 4). The increased flexibilities in Leu253-Cys282 and Ala329-Phe380 regions of FZD6 mutant could be explained by the crosstalk between these regions from loop structures, closely located to each other (Additional file 1: Figure S1). It is also important to notice that KTxxxW motif, considered as significant for FZD signaling, is not closely located to these listed regions. Specifically, we focus on the flexibilities of residues in the KTxxxW motif. As displayed in Additional file 1: Figure S3, the increase in flexibilities of KTxxxW motif has been captured in mutant protein. This particular increase in KTxxxW motif flexibility could be considered as a part of the general trend observed in the mutant protein with respect to native one.

**Fig. 5:**
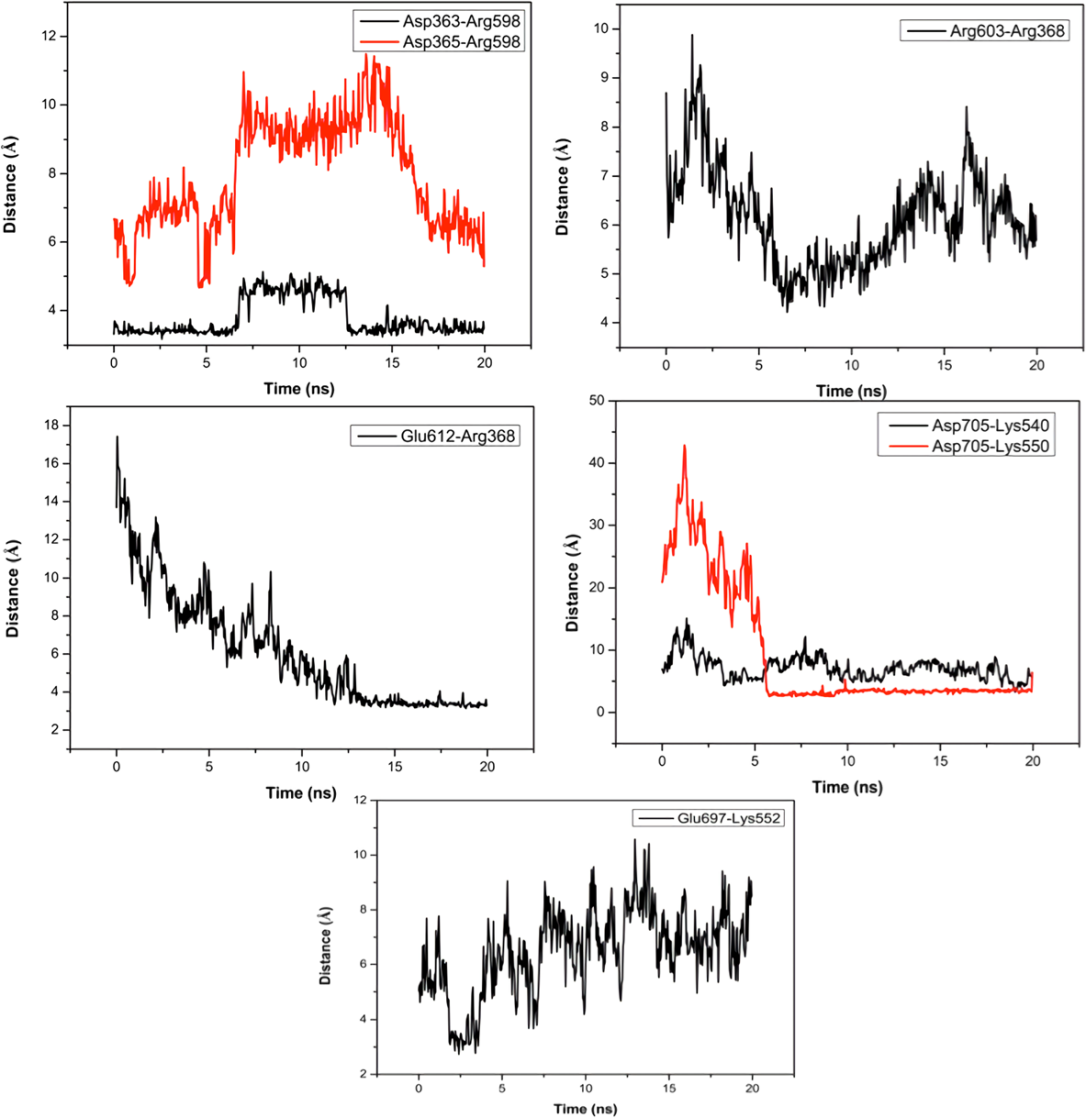
The salt bridge interactions loss in FZD6 upon mutation

Secondly, the significant salt bridge interactions for native and mutant proteins are evaluated along 20 ns MD trajectory. Due to the existence of p.Gly559Aspfs*16 mutation, 28 salt bridge interactions among 89 ones do not exist in FZD6 mutant proteins. This number corresponds to almost 30% of all salt bridge interactions, and the loss of this portion would be one of the reasons behind increased RMSD pattern (Fig. 4a). Among these 28 salt bridge interactions, seven are established between C-terminal structure and β–sheet structures, being considered as a part of the seven transmembrane-spanning receptors (Fig. 1). Thus, the loss of these particular interactions results in the weakening of intra-molecular interactions in an obvious way. As displayed in Fig. 5, these salt-bridge interactions are mostly strong, except Glu697-Lys552, to contribute the stability of the protein in a positive manner.

## Discussion

We report here a consanguineous Turkish family with three affected individuals having homozygous 8 bp truncation mutation, p.Gly559Aspfs*16. The mutation we found was previously reported in two other Turkish families, indicating founder effect. The phenotype of the affected individuals in our family is very similar to the other two families; except the uveitis in the index patient. The diagnosis of uveitis and possible ocular tuberculosis in the index patient is noteworthy. Nine months of anti-tuberculosis agents were given to the index patient and, for the first 2 months, corticosteroids were also used.

Fröjmark et al. were the first to link mutations in *FZD6* gene to autosomal recessive nail dysplasia [3]. They identified two different mutations in two large consanguineous Pakistani families. Affected individuals were homozygous for the missense mutation p.Arg511Cys for one family and homozygous for the nonsense mutation p.Glu584Ter in the other family. Later, several reports of other patients from Pakistan, Iran, and Turkey were reported. Naz et al. reported two more Pakistani families where affected individuals were also homozygous for mutation p.Glu584Ter, indicating a common ancestor [4]. Raza et al. also reported another Pakistani family with a homozygous p.Gly422Asp; c.1265G > A mutation [5]. At the same year, two other families with new mutations were reported; in one family affected individuals were homozygous for missense mutation p.Arg509Ter and in the second family affected individuals were compound heterozygous for mutations p.Arg96Cys/p.Glu438Lys [34]. Moreover, in 2016, homozygosity for an 8 bp deletion, p.Gly559Aspfs*16; c.1676_1683delGAACCAGC was detected in two Turkish families [8]. In 2017, a homozygous 1 bp deletion variant, c.1859delC (p.Ser620Cysfs*75) was seen in an Iranian family [7]. To date, seven different mutations have been reported in eleven families, including two missense, two nonsense, two frameshifts, and one compound heterozygous [3–8]. Five out of seven mutations are clustered in the C-terminus, which suggests that the C-terminal region could be a mutation hotspot.

Through mutagenesis studies, it has been revealed that several residues in the intracellular loops and the C-terminus of FZDs play critical roles for signaling. Specifically, the mutation of the highly conserved internal KTxxxW motif between 498th and 503rd positions in the C-terminus, or single amino acid exchanges in the first (R340A) or the third (L524A) intracellular loops of, another protein from human FZD family, FZD5 completely abolished FZD signaling. The same mutations completely ablated the binding of the phosphoprotein DVL and its membrane recruitment by FZD [18] which is a central player in FZD-induced signal transduction and functionally necessary for all FZD signaling pathways [35, 36]. The PDZ domain of DVL directly binds the KTxxxW motif of FZD [37].

Also, in general, agonists binding to GPCRs were shown to induce changes in C-tail conformation that is necessary for activating heterotrimeric G protein [38]. Prolonged agonist stimulation catalyzes the phosphorylation of the C-tail, promoting arrestin binding, desensitization, and GPCR internalization [39]. Studies utilizing the peptides encoding the C-tail of FZD also suggest alpha-helicity in C-terminus of the Frizzleds is known as a need for efficient protein-protein interaction with DVL and other downstream signaling elements. Moreover, shortening the C-tail beyond C507 of, another protein from human FZD family, FZD5 impaired regular DVL recruitment and the ability of Wnt activator to activate Lef/ Tcf-dependent transcription [40, 41].

In terms of experimental studies, the existing knowledge about FZD6 is still limited. Fröjmark et al. expressed native and mutant (p.Glu584X and p.Arg511Cys) variants of FZD6 fused to green fluorescent protein (GFP) in HEK293T cells. While the missense mutation has no or little effect on total FZD6 levels, no expression was detected from the nonsense one [3]. The biological function of FZD6 protein was also studied by Cui et al., using a FZD6 knock-out mice, and their findings pointed out a regulatory role for FZD6-mediated Wnt signaling in the differentiation process of claw/nail formation [42]. Moreover, FZD6 was shown to be critical for the morphogenesis of hair follicles in *Drosophila* and mice. Fzd6−/− mice are viable and fertile; but among more than 100 FZD6 knock-out mice examined, all have abnormal macroscopic hair whorls [43]. Besides, FZD3−/− and FZD6−/− double-mutant mice die within minutes of birth and have a misoriented pattern of inner-ear sensory hair cells, this points out the role for FZD6 in planar-cell polarity. Also, FZD6 genes are expressed in all sensory hair cells and in many non-sensory epithelial cells in the inner ear [44]. Moreover, Fröjmark et al. reinvestigated the FZD6 knock-out mouse model and about 50% of male knock-out mice, but none of the female mice had absent or abnormal claws compared to wild-type mice. To link the expression of FZD6 to early nail development they also checked FZD6 expression in mouse embryos at several embryonic days and revealed that at E16.5 there was an expression of FZD6 in the epidermis of the digital tip in the region corresponding to the developing nail bed and ventral part of the digit [3]. In addition to that, Naz et al. reported a strong expression level of FZD6 in the ventral nail matrix and some FZD6 staining in the nail bed [6].

So far, ten types of NDNC have been reported in the literature, 6 of which are inherited in an autosomal dominant mode of inheritance. The genes associated with human hereditary nail disorders are listed as *HPGD, RSPO4, PLCD1, COL7A1* and *FZD6* [2]. *HPGD* gene is found to be associated with isolated congenital nail clubbing (OMIM 119900) and responsible for the metabolism of prostaglandins. Following irritation or injury, arachidonic acid (AA) is released and oxygenated by calcium-dependent enzyme systems leading to the formation of prostaglandins. Specifically prostaglandin E2 is readily detectable in equine acute inflammatory exudates. Moreover, both the influx of extracellular calcium and mobilization of intracellular calcium are very critical for the process of prostaglandin formation [45]. Another gene is *RSPO4*, linked to nail disorder, nonsyndromic congenital (NCDC4; OMIM 206800); a secreted protein with a known role in embryonic development and homeostatic self-renewal in adult tissues; besides its role in Wnt signaling which has both anti-inflammatory and pro-inflammatory functions. *PLCD1* is linked to NDNC3 (OMIM 151600); a member of the phospholipase C family that regulates homeostasis of the immune system in skin. The lack of PLCD1 protein induces skin inflammation; since the skin of PLCD1-ko mice displays typical inflammatory phenotypes, including increased dermal cellularity, leukocyte infiltration and expression of pro-inflammatory cytokines. In addition, exogenously expressed PLCD1 attenuates LPS-induced expression of IL-1b [46]. Another gene related with nail disorders is COL7A1, which the alpha chain of type VII collagen that is associated with NDNC8 (OMIM 607523). Mutations in COL7A1 induce lifelong severe skin and mucosal blistering followed by scarring, caused by loss of adhesion between the epidermis and the dermis. COL7A1-ko mice also display blisters and erosion at sites of trauma, subepidermal blistering, and high postnatal lethality. Finally, FZD6 function as a negative regulator of the canonical Wnt/beta-catenin signaling. It was observed that FZD6 signalling activates beta-catenin in a study of patients affected by nail dysplasia. This study reported that Wnt3a signalling causes beta-catenin accumulation in healthy, but not FZD6-mutant fibroblasts, indicating a canonical role of FZD6 in this context [3]. Moreover, Kilander et al. showed via fluorescence recovery after photo-bleaching (FRAP) that recombinant WNT-1, − 2, 3A, − 4, −5A, −7A, -9B, and -10B affect FZD6 surface mobility and thus directly act on FZD6 [47]. The loss of interaction partners we proposed due to our truncation mutation could mainly be WNT family proteins. WNT pathway and innate immunity are also shown interrelated. There is an interaction among the WNT signaling network, inflammatory cytokines, and innate immune signaling pathways [48]. Individual WNT proteins were shown to have pro- or anti-inflammatory functions. WNT ligands and WNT/β-catenin signaling was found to positively regulate LPS-induced pro-inflammatory cytokines. The WNT signaling pathway plays a major role in regulating tolerance versus immunity, particularly in DCs, and more [49]. Therefore, it is not unexpected that immune-related problems are seen in NDNC patients. The common intersection point of all known NDNC genes is their association with the immune system, specifically innate immunity [50, 51]. The diagnosis of uveitis and possible ocular tuberculosis in the index patient is noteworthy as the innate immune system is a rapidly deployed, first response in host defense and its dysregulation leads to autoinflammation. Ocular follow-up of our patient will probably help differentiate between an auto-inflammatory granulomatous process and ocular tuberculosis.

Due to the lack of crystal structure of FZD6, computer-based analyzes of FZD6 are also very limited. The first attempt made by Mohammadi-asl, et al. as predicting the formation of multiple helical secondary structures in the distal cytoplasmic region of the p.Ser620Cysfs*75 mutant protein which does not exist in the native protein via I- TASSER [52]. Moreover, they used NtePhos 3.1 server and revealed pathogenic consequence of the mutation by disturbing the cytoplasmic domain structure and signaling through loss of phosphorylation residues [7]. They concluded that the nonsense mutation causes the elimination of the distal end of the topological domain (amino acids 495–706). This domain mediates Wnt/beta-catenin signaling by relocalization and phosphorylation of disheveled proteins. Even though, KTXXXW is present, loss of phosphorylation residues and formation of unusual helical secondary structures can result in lack of proper response to WNT-3A and WNT-5A activation consistent with previous studies [3, 4, 7].

For the same reason, we performed the homology modeling of native and mutant forms of FZD6 protein with I-TASSER. To gain more insight about the impacts of mutation on the structure of the protein, we performed 20 ns MD simulations and concluded that FZD6 mutant displayed higher RMSD pattern compared to native. This result suggests us that the introduction of stop codon to C-terminus, associated with the translation of new 15 amino acids upon the frame-shift, results in increased tendency for unfolding than native structure with higher backbone motion at 310 K. This result is also supported with RMSF pattern that Leu253-Cys282, Ala329-Phe380 and His549-Ser571 regions (mutant numbering) are more flexible in FZD6 mutant compared to native one. Specifically, His549-Ser571 region (mutant numbering) displays almost ~ 8-fold more flexibility compared to native. Hence, the loss of C-terminus, even being in partial, would disrupt the intramolecular interactions and we could end up with unstable protein. It is also crucial to notice that the conservation of KTxxxW motif in C-terminus; previously suggested as essential for FZD signaling in, another protein from human FZD family, FZD5 protein, is not enough for the FZD6 mutant. This fact suggests that the problem in our case can be the loss of structural integrity in addition to the loss of signaling region.

Along 20 ns MD trajectory, we also consider the impacts of mutations in terms of intramolecular interactions such as salt-bridge formations. Upon this particular mutation, almost 30% of salt-bridge interactions are lost. Even explaining protein stability is a complex issue, there is a well-known fact in literature that the intra-molecular interactions, such as salt bridge formations, are crucial elements for the stability of proteins and their positions on 3D structure of protein contribute to its stability in different manners, e.g. the salt-bridge formations on protein surface contribute to protein stability less than 1 kcal/mole [53] while those buried and positioned on hydrophobic core contribute more than 4 kcal/mole [54]. The loss of these seven salt bridge formations, established with β–sheet structures, considered as a part of the seven transmembrane-spanning receptor (Fig. 1), would adversely affect the protein stability and result in its non-functionality. Except for Glu697-Lys552 interactions, these particular salt bridge interactions are strong enough to contribute to the stability of the protein in a positive manner. As a well-known fact, entropy is a crucial element of thermodynamics of macromolecules to create a favorable environment for protein or substrate binding, happened in the signaling pathway. Upon the alterations in entropy of protein with negative manner caused by the loss of these salt bridge interactions, the expected interaction(s) of protein would be either lacked or disrupted in FZD6 mutant and this non-functionality happens.

## Conclusion

In summary, we identified a consanguineous Turkish family with NDNC10 via the identification of a homozygous frameshift mutation, p.Gly559Aspfs*16, in *FZD6* gene. Published functional data of FZD family proteins convincingly demonstrate the importance of C-terminus on signal transduction of FZDs. The 8 bp deletion reported leads to loss of 133 amino acid in C-terminus of FZD6 compared to native protein. This study contributes to the existing knowledge by proposing that the pathogenicity of this frameshift mutation is caused by disturbing the C-terminal domain structure and hence interaction partners of FZD6. Further research is needed to thoroughly understand the FZD6 function and its role in innate immunity in association with nail dysplasia.

## Additional file

Additional file 1: **Figure S1**. The secondary structure formations in FZD6_mut displaying higher RMSF values compared to native enzyme. **Table S1**. *FZD6* mutations that are found to be associated with NCDC10. **Figure S2**. Paralogs of FZD6 protein. **Figure S3**. Comparison of the flexibilities of residues in KTxxxW motif (DOCX 2076 kb)

## Abbreviations

AA: Arachidonic Acid
BWA: Burrows-Wheeler Aligner
CADD: Combined Annotation Dependent Depletion
CRD: Cysteine-Rich Domain
ExAC: Exome Aggregation Consortium
GATK: Genome Analysis Toolkit
GnomAD: Genome Aggregation Database
M-CAP: Mendelian Clinically Applicable Pathogenicity
NDNC 1–10: Nail disorder, nonsyndromic congenital 1–10
Polyphen: Polymorphism Phenotyping
REVEL: Rare Exome Variant Ensemble Learner
RMSD: Root-Mean-Square Deviation
RMSF: Root-Mean-Square Fluctuation
SIFT: Sorting Intolerant From Tolerant
WES: Whole Exome Sequencing
