# Supplementary material for: A possible founder mutation in FZD6 gene in a Turkish family with autosomal recessive nail dysplasia
Source: BMC Med Genet. 2019 Jan 14;20:15. doi: 10.1186/s12881-019-0746-6 (PMC6332616; doi:10.1186/s12881-019-0746-6)
Supplement: Supplementary file 1 — Figure S1. The secondary structure formations in FZD6_mut displaying higher RMSF values compared to native enzyme. Table S1. FZD6 mutations that are found to be associated with NCDC10. Figure S2. Paralogs of FZD6 protein. Figure S3. Comparison of the flexibilities of residues in KTxxxW motif (DOCX 2076 kb) [file 12881_2019_746_MOESM1_ESM.docx]

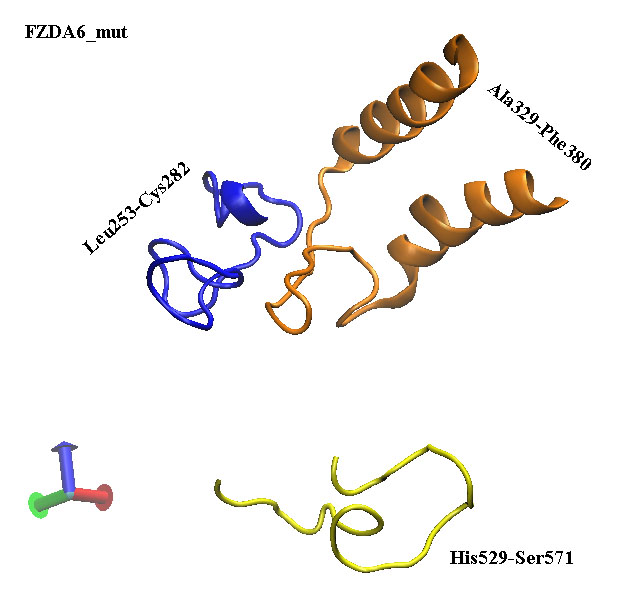


**Supplementary Fig 1.** The secondary structure formations in FZD6 mutant displaying higher RMSF values compared to native enzyme

| **Amino acid Change** | **Mutation Type** | **Mode of Inheritance** | **Location** | **Ref** |
| --- | --- | --- | --- | --- |
| Gly422Asp | Missense | Homozygous | 6^th^ transmembrane domain | Raza *et al.,* 2012 |
| Arg509Ter | Nonsense | Homozygous | C-terminal | Wilson *et al.,* 2013 |
| Arg511Cys | Missense | Homozygous | C-terminal | Fröjmark *et al.,* 2011 |
| Gly559Aspfs*16 | Frameshift | Homozygous | C-terminal | Kasparis *et al.,* 2016 |
| Glu584Ter | Nonsense | Homozygous | C-terminal | Fröjmark *et al.,* 2011 |
| Ser620Cysfs*75 | Frameshift | Homozygous | C-terminal | Mohammadi-asl *et al.,* 2017 |
| Arg96Cys/Glu438Lys | Missense | Compound Heterozygous | N-terminal/3^rd^ extracellular loop | Wilson *et al.,* 2013 |

**Supplementary Table 1.** *FZD6* mutations that are found to be associated with NCDC10


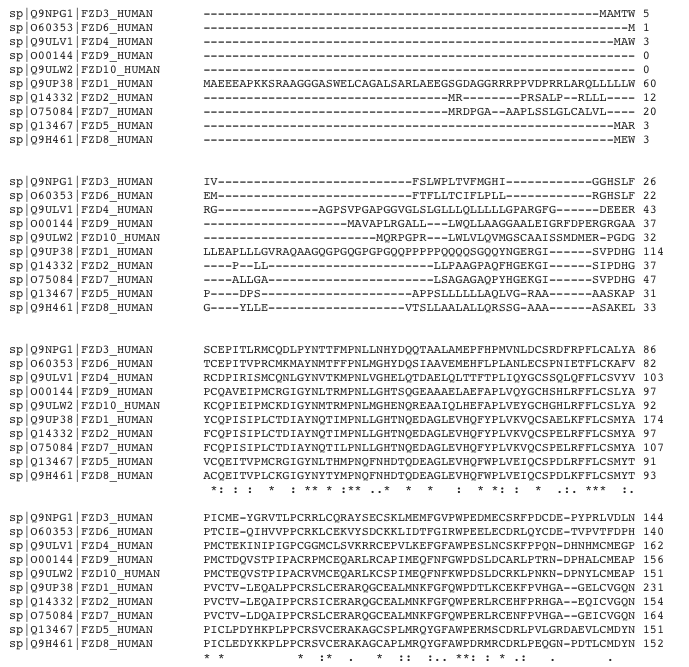


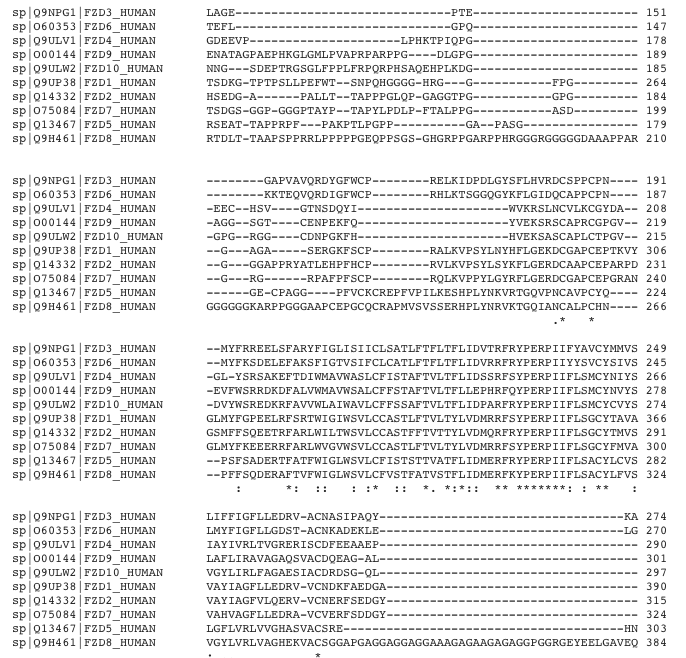


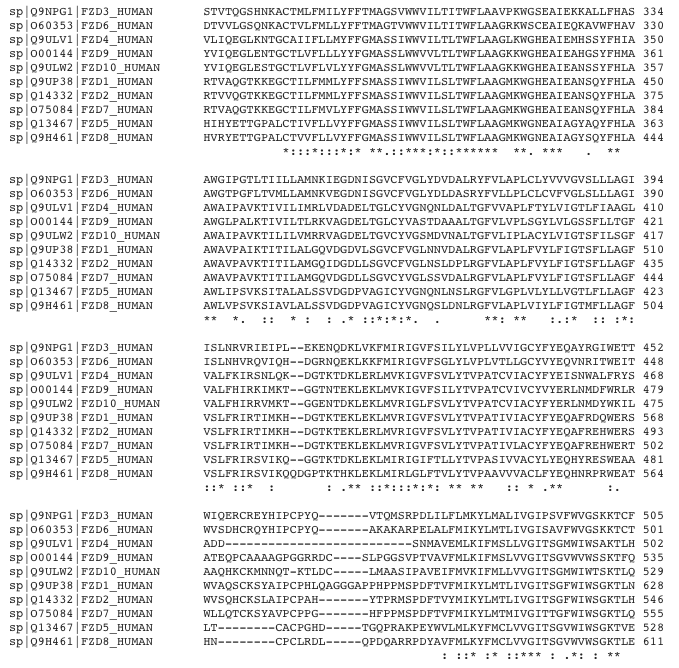


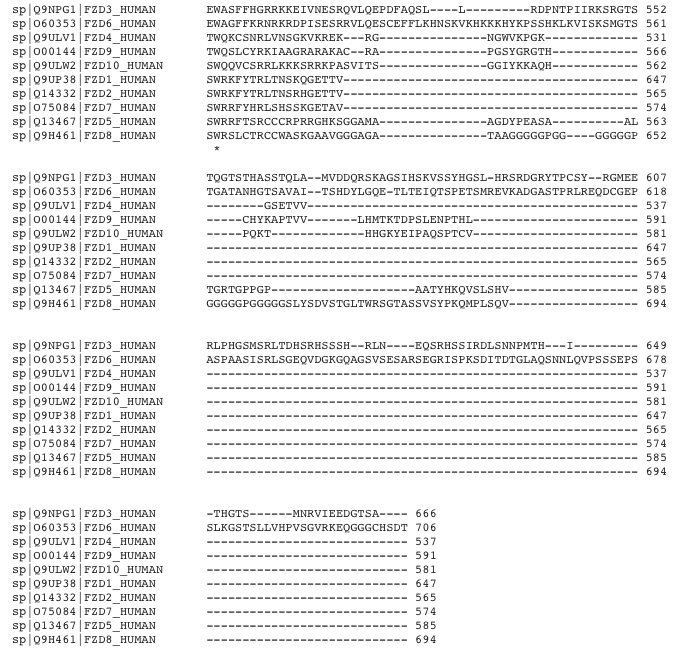


**Supplementary Fig 2.** Paralogs of FZD6 protein


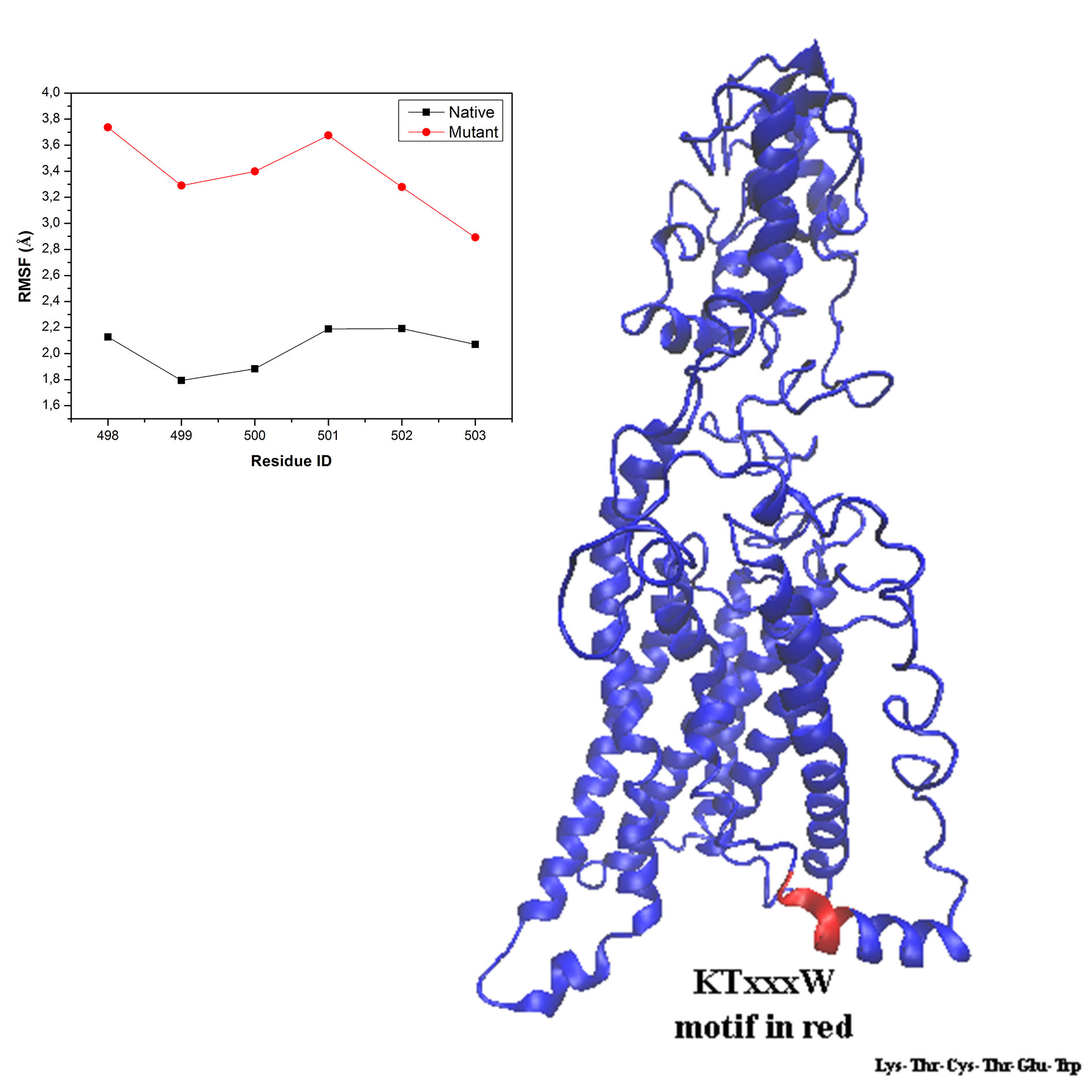


**Supplementary Fig 3.** Comparison of the flexibilities of residues in KTxxxW motif
